# The connection between climate change and rising river drowning incidents

**Published:** 2024-10

**Authors:** Seyed Ahmad Mohammadi Kia, Yahya Saleh Tabari, Mehdi Bagheri

**Affiliations:** ^*a*^ Department of Health in Disasters and Emergencies, Shahid Sadoughi University of Medical Sciences, Yazd, Iran.; ^*b*^ Emergency Medical Services, Mazandaran University of Medical Sciences, Sari, Iran.; ^*c*^ Babolsar Health Network, Mazandaran University of Medical Sciences, Sari, Iran.

## Abstract

**Background:**

Climate change is significantly impacting natural environments, leading to rising river levels and more frequent and severe floods. This paper explores the direct link between climate change and the increase in river drowning incidents. It examines how changes in water ecosystems, socio-economic disparities, and infrastructure contribute to heightened drowning risks.

**Methods:**

The study used a comprehensive review of existing literature and data on climate change, water safety, and drowning incidents. It evaluates the effects of rising temperatures, extreme weather events, and socio-economic factors on public safety in aquatic environments. Additionally, it assesses current preventive measures and proposes strategies for mitigating these risks.

**Results:**

Rising temperatures and changes in precipitation patterns are causing higher river levels and increased flooding, leading to more drowning incidents. The study highlights how socio-economic inequalities, such as lower income and a lack of access to swimming lessons, exacerbate these risks. Migrants and displaced persons face additional dangers due to unfamiliarity with local water hazards and increased exposure to natural disasters. Effective prevention requires a combination of public awareness campaigns, safety infrastructure improvements, and targeted interventions for vulnerable groups.

**Conclusions:**

Urgent action is needed to address the intersection of climate change and water-related public safety. Building resilient infrastructure, increasing public awareness, and implementing socio-economic interventions are critical to reducing drowning incidents. Policymakers, communities, and individuals must collaborate to navigate the complex landscape of climate change and its effects on water safety. By adopting adaptive and innovative approaches, it is possible to significantly mitigate drowning risks and save lives for future generations.

**Keywords:**

Climate change, River drowning incidents, Water safety, Flooding, Public awareness

